# COVID-19 Infection in Kidney Transplant Patients: An Italian One Year Single Centre Experience

**DOI:** 10.3390/pathogens10080964

**Published:** 2021-07-30

**Authors:** Mariarosaria Campise, Carlo Maria Alfieri, Marta Perego, Francesco Tamborini, Donata Cresseri, Maria Teresa Gandolfo, Valentina Binda, Anna Regalia, Piergiorgio Messa

**Affiliations:** 1 Department of Nephrology, Dialysis and Kidney Transplantation, Fondazione IRCCS Ca’ Granda Ospedale Maggiore Policlinico, 20122 Milan, Italy; carlo.alfieri1@gmail.com (C.M.A.); marta.perego4@studenti.unimi.it (M.P.); francesco.tamborini@unimi.it (F.T.); donata.cresseri@policlinico.mi.it (D.C.); mariateresa.gandolfo@policlinico.mi.it (M.T.G.); valentina.binda@policlinico.mi.it (V.B.); anna.regalia@policlinico.mi.it (A.R.); piergiorgio.messa@unimi.it (P.M.); 2 Department of Clinical Sciences and Community Health University of Milan, 20122 Milan, Italy

**Keywords:** COVID-19, kidney transplantation, immunosuppressive therapy

## Abstract

COVID-19 is a life-threatening infection among elderly patients, comorbid patients, or transplanted patients. Lombardy (region of Italy), accounts for 786,324 cases as of 21 April 2021. We retrospectively describe our single Centre experience in 82 adult kidney-transplant patients with COVID-19 infection during two pandemic outbreaks: 27 (first outbreak) and 65 (second outbreak). Thirty-seven patients were hospitalized (HP) and sixty-five were home managed (HM). Infection presented with fever (80%), cough (51%), and dyspnea (33%). HP were older (60 ± 11 vs. 50 ± 14 years, *p* = 0.001), had more severe respiratory symptoms (dyspnea 62.1%, *p* < 0.0001–cough 67% *p* = 0.008), and a longer length of disease (30 ± 28 vs. 21 ± 10, *p* = 0.04). The incidence of acute kidney injury (AKI) was 29.7% (*p* < 0.0001). Steroid dosage was increased in 66% of patients (*p* = 0.0003), while calcineurin inhibitors were reduced by up to one third in 45% of cases, *p* < 0.0001. Eleven patients died (13%). HM patients recovered completely without sequelae. In the overall cohort, AKI development (*p* = 0.006 OR 50.4 CI 95% 3.0–836) and age (*p* = 0.04 OR 1.1 CI 95% 1.0–1.2) were the most important factors influencing the probability of death during the infection. Although we report a relatively low incidence of infection (5.1%) the incidence of death is almost four times higher than it is in the general population.

## 1. Introduction

Corona viruses are enveloped RNA viruses broadly distributed among humans, other mammals, and birds. They cause respiratory, enteric, hepatic, and neurologic diseases. Six coronavirus species are known to cause human disease [1]. Four viruses (229E, OC43, NL63, and HKU1) are prevalent and typically cause common cold symptoms in immunocompetent individuals [2]. The two other strains, Severe Acute Respiratory Syndrome CoronaVirus (SARS-CoV) and Middle East Respiratory Syndrome CoronaVirus (MERS-CoV), are zoonotic in origin and have been linked to occasionally fatal illnesses. The COVID-19 illness was first reported in December 2019 in a cluster of patients with pneumonia of unknown cause and was linked to a seafood wholesale market in Wuhan, China. After the first Chinese description, the infection spread all over the world acquiring pandemic characteristics [3]. Italy was the first most affected country in Europe, with a total of 3.9 Million cases as of 21 April 2021. Of them, 118,000 people died, 3.31 Million recovered from the infection and 475,635 still attested positive with a nasopharyngeal swab for SARS-CoV-19 [4]. The clinical characteristics of COVID-19 mimic those of SARS-CoV. Fever and cough are the dominant symptoms. Gastrointestinal symptoms (including taste and smell dysfunction) are less common. In a minority of patients, fever can be absent; therefore, afebrile patients may be missed and non-diagnosed with the infection. Lymphocytopenia is common and, in some cases, severe. Generally, symptoms appear after an incubation period of approximately 5 days [5]. The worsening of symptoms may lead to death in a median of 14 days [6]. Evolution to death depends mainly on age, comorbid condition, and the patient’s immune system status. For this reason, kidney transplanted patients are considered at high risk for developing a fatal infection. The aim of this study is to describe our one-year single centre experience in managing kidney transplant patients with COVID-19 infection.

## 2. Materials and Methods

Ninety-four patients out of 1588 followed at our centre reported symptoms compatible with the COVID-19 infection from 1 March to 30 April 2020 (first outbreak, 30 patients) and from 1 September 2020 to 21 April 2021 (second outbreak, 64 patients). Eighty-two of them were included in this analysis. The reasons for missed inclusion were a COVID negative nasopharyngeal swab or insufficient information either on some relevant variables or on outcome.

The diagnostic criteria for a suspicion of COVID-19 infection were the following: possible contact with another person in whom COVID-19 infection was confirmed, the presence of fever, respiratory symptoms, diarrhea, and taste and smell dysfunction in a length of time compatible with the estimated incubation period. Diagnosis of certainty was based on the positivity of the RNA test via nasopharyngeal swab and/or the presence of typical computed tomography findings such as ground glass opacities particularly on the peripheral and lower lobes. Patients with severe or worsening respiratory symptoms suspected for COVID-19 pneumonia were referred to the hospital nearest to them. Patients with other symptoms but without the respiratory syndrome were treated at home.

When a health emergency was declared, we implemented preventive measures mainly regarding outpatient management. We extended the interval of time between visits in the presence of stable renal function to reduce the number of hospital accesses. If the transplant vintage was below 6 months, visits were confirmed without accompaniment unless the patient had a disability. A dedicated phone number available 24 h a day, 7 days a week was given for immediate assistance and eventual hospital admission when needed.

Since the start of the pandemic our guidelines on treatment of infection, included the immediate withdrawal or reduction of the anti-proliferative agent in use associated with the increase of prednisone dosage up to 1 mg/kg/day. This protocol was modified based on emerging indications so that the steroid dose was further increased, and the patients were switched to dexamethasone at the dose of 6 mg/day for 10 days either orally or intravenously, depending upon the patient’s condition. The presence of severe acute kidney injury (AKI) was defined as a renal replacement therapy need due to a significant worsening of the renal function (serum creatinine increase >2 times) in two consecutive measurements and/or a severe urine output reduction (urine output < 0.5 mL/kg/h for >12 h) or anuria for 12 h. The recovery day was the day symptoms ended or the patient was discharged from hospital. Patients returned to the outpatient’s clinic after two negative nasopharyngeal swabs were performed one week apart.

All procedures performed in this study involving human participants were in accordance with the ethical standards of the institutional and/or national research committee and with the 1964 Helsinki declaration and of the Declaration of Istanbul and its later amendments or comparable ethical standards.

### Statistical Analysis

In the statistical analysis continuous variables were expressed as median value and interquartile range (25%ile–75%ile), and nominal variables were reported as number of cases (n) and relative percentage.

Differences among groups were determined by Student’s *t* test and Kruskal-Wallis test and differences among percentages were determined by χ^2^ or Fisher test if appropriate.

Logistic multiple regressions were used to perform multivariate analysis. Statistical analyses were performed using software Statistica version 10 and MedCalc. Significance was set for *p* values < 0.05.

## 3. Results

### 3.1. Patients Characteristics at COVID-19 Onset

At our Institution, we followed 82 consecutive adult kidney transplant recipients who had the COVID-19 infection between 1 March and 30 April 2020 (first outbreak, 27 patients) and from 1 September 2020 to 21 April 2021 (second outbreak, 55 patients). Fifty-four were male (65.8%), the median age was 55 years (range 24–83) and the median transplant vintage was 118 months (39–229). Sixty-five patients received a deceased donor kidney transplant (79.2%) and seventeen received a living donor one. The prevalence of hypertension and insulin dependent diabetes was 81.7% and 32.1%, respectively. Inhibitors of the Renin–Angiotensin system (iRAS) were used in 25.6% of patients. One patient had peripheral artery disease, 20.8% had heart disease (aortic valvular replacement, hypertensive heart disease, coronary artery disease, paroxysmal atrial fibrillation), and one patient had chronic pulmonary obstructive disease. In eight patients, a diagnosis of non-melanoma skin cancer was made during the transplant follow-up before the infection. Another patient underwent an endoscopic removal of a high-grade left colon dysplasia. Two patients had active cancer at the time of the COVID-19 infection: one had a post-transplant lympho-proliferative disease (diffuse large B-cell Lymphoma) and the other had breast cancer.

The immunosuppressive therapy included prednisone, calcineurin inhibitors (CNI), and mycophenolate mofetil (MMF) in 74, 80 (18 Cyclosporine, 63 Tacrolimus), and 55 cases, respectively. Everolimus and rapamycin were used combined with tacrolimus and with mycophenolate, respectively, in six patients. In both cases, the reason was minimizing or avoiding CNI because of repetitive surgery for non-melanoma skin cancer.

Most patients had a normal renal function: median plasma creatinine 1.46 (1.2–1.8) mg/dL, with mild or absent proteinuria: median 0.16 mg/day. One case of biopsy proven IgA nephritis recurrence and two transplant glomerulopathy, accounted for proteinuria above 1 g/24 h in three patients. The median hemoglobin level was 12.9 g/dL.

The origin of Covid-19 contagion was unknown in 70.7% of cases. Twelve patients were infected by a family member, nine were exposed to the contagion at work, and three were exposed during an access to the hospital. The most common initial symptom was fever usually above 37.5 °C in 80.4% of cases and respiratory symptoms: cough and/or dyspnea in 51.2% and 32.9% of cases, respectively. Ten patients (12.1%) reported smell and taste dysfunction. In five patients (6%), diarrhea was the first symptom, followed by fever and respiratory symptoms. Symptoms lasted for a median time of 21 days.

Thirty-seven patients (45%) with a rapid evolution of respiratory symptoms with overt dyspnea and reduced oxygen saturation were immediately referred either to our hospital or to the nearest hospital and transferred to ours upon availability of a hospital bed: 12 during the first outbreak and 25 during the second. The remaining 45 patients with milder symptoms were treated at home. (Table 1).

#### 3.1.1. Hospitalized Patients

The principal differences between hospitalized and non-hospitalized patients are reported in Table 2 and Table 3. Compared to non-hospitalized patients, those who were hospitalized were older (60 ± 11 years vs. 50 ± 14 years, *p* = 0.001), had more severe respiratory symptoms (dyspnea, 62.1%, *p* < 0.0001—cough, 67% *p* = 0.008), and had a longer length of disease (30 ± 28 vs. 21 ± 10, *p* = 0.04). AKI was found only in hospitalized patients (29.7%) (*p* < 0.0001).

Regarding chronic immunosuppression, patients did not differ significantly. The majority of them were treated with a triple therapy with CNI; therefore, no significant difference in the outcome could be detected.

As for therapy modification, an increase in steroid dosage was prescribed in 66% of patients, *p* = 0.0003 while a CNI reduction up to one third of the original dosage, was done in 45% of patients, *p* < 0.0001. A total of 11 patients died (13%).

#### 3.1.2. First Outbreak (From 1 March to 30 April 2020)

Patients hospitalized during the first outbreak had a significantly longer transplant vintage than those who were not hospitalized: 210 ± 108 months vs. 75 ± 86 months, *p* < 0.0001. Dyspnea was significantly more frequent, 66% (*p* < 0.001), as was AKI, present in 50% of patients (*p* = 0.03). Steroid dosage was increased in 41% of patients (*p* = 0.18) and CNI reduction up to one third of the original dosage was conducted in 50% of them, *p* = 0.003.

Five patients died. They had more severe respiratory symptoms (dyspnea 80%, *p* < 0.001 and cough 91% *p* = 0.09), and AKI was found in four of them (80%, *p* < 0.001).

Six patients were treated with non-invasive ventilation using a continuous positive airway pressure helmet, while five required immediate tracheal intubation. Computed tomography (CT) findings showed ground glass opacities, particularly on the peripheral and lower lobes and bilateral multiple lobular areas of consolidation in nine patients in whom the investigation was conducted. On admission, the nasal swab was positive in 10 patients and was not performed in two in whom Bronchoalveolar Lavage (BAL) was performed.

At the beginning of the pandemic, patients with critical status were hospitalized as near as possible to their residence, making a homogeneous pharmacological approach very difficult. Immunosuppression modification included: MMF withdrawal (seven patients), CNI withdrawal (six patients), and steroid dosage increasing by up to 1 mg/kg/day (five patients). Anti-coagulation therapy was used in all the patients. Inhibitors of the Renin–Angiotensin System were withdrawn whenever present. Hydroxychloroquine was used in four patients, while anti-viral treatment the lopinavir/ritonavir association was administered in five patients. One patient developed hepatic toxicity and was shifted to darunavir. Finally, in one patient, the anti-interleukin-1 receptor antagonist anakinra was used.

Six out of 27 patients died (22%). One patient, with a history of aortic valve replacement, died 24 h after hospitalization for acute respiratory insufficiency. Four more patients died within 1 month since the onset of symptoms. All of them had AKI and three required dialysis treatment. Only one patient who also had AKI requiring dialysis cleared the infection and was discharged from ICU 17 days later with a restored renal function: last plasma creatinine 1.5 mg/dl. The other four patients recovered without respiratory impairment.

#### 3.1.3. Second Outbreak (1 September 2020 to 21 April 2021)

With the second outbreak we tried—whenever possible—to admit patients to our hospital or to other hospitals referring to us as Transplant Centre to achieve more homogeneous management. Only two patients out of 25 were hospitalized outside of Lombardy close to their residence. Of the remaining patients, one was treated with a high flow ventilation mask, three were treated with non-invasive ventilation using a continuous positive airway pressure helmet, and one required tracheal intubation.

Patients hospitalized during the second outbreak were older (61 ± 11 vs. 50 ± 14 years, *p* = 0.004), had more frequently respiratory symptoms (dyspnea 60%, *p* < 0.0001), and had a longer length of disease (36 ± 31 vs. 23 ± 14, *p* = 0.03). AKI developed in 20% of patients (*p* = 0.01). Increased steroid dosage together with a reduction of CNI was done in 76% and 44% of patients (*p* = 0.001 in both cases).

During the second outbreak 6 patients died. They had more severe respiratory symptoms (dyspnea 83%, *p* = 0.008 and cough 56%, *p* = 0.05). In this group of patients, the incidence of AKI was 33%, *p* = 0.08. 

Upon admission, the immunosuppressive treatment was CNI inhibitor based in all 25 patients, while two of them were steroid free and six had a double therapy avoiding MMF for side effects. Therapy modification included immediate MMF withdrawal and possibly a reduction to one-third of the original CNI dosage. Based on the new protocol, patients were switched to dexamethasone at the dose of 6 mg/day for ten days either orally or intravenously depending on the patient’s condition.

**Figure 1:**
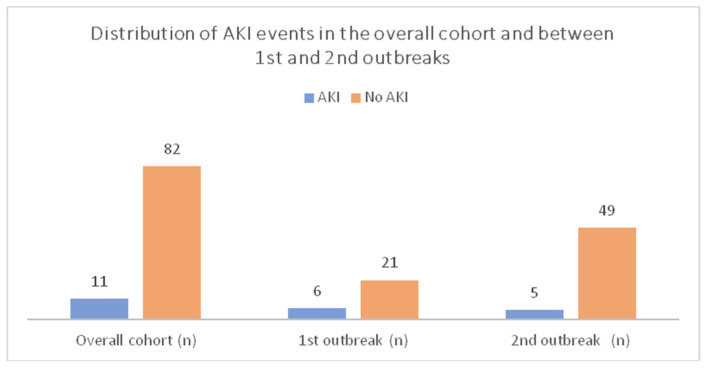
AKI distribution in the overall cohort and between 1st and 2nd outbreaks.

Five patients developed AKI. One of them (whose plasma creatinine was above 2.5 mg/dl at the time of infection) started chronic hemodialysis. Figure 1 presents the graphical distribution of AKI in the overall cohort between the 1st and 2nd outbreaks. Nineteen patients recovered without either respiratory or renal sequelae. Mortality was reduced to 9% (5 patients). Of note, three of them died within 48 h upon hospitalization.

#### 3.1.4. Home Managed Patients

Fifty-five patients with a less critical disease were managed at home. From the day they first reported symptoms possibly related to the COVID-19 infection to the day they were declared cured, patients received a daily phone call by one of the transplant physicians.

Question around general wellbeing and symptoms’ evolution with attention to respiratory symptoms, if any, were asked. Since symptom severity was milder in home-managed patients, treatment consisted mainly of immunosuppressant reduction. MMF and Everolimus were completely withdrawn in all patients. No modification of CNI dosage was made. Prednisone was increased up to 25 mg/day in 11 patients and no anti-thrombotic therapy was administered. Before the symptoms were clearly related to the COVID-19 infection, six patients had been treated with antibiotics: fluoroquinolones (two patients), azithromycin (two patients), and third generation cephalosporin (two patients). Of note, the prescription was made by the general physician contacted by the patients before calling the Transplant Centre. Two patients—both during the first outbreak—were treated with hydroxychloroquine. One of them, a young woman transplanted for end stage renal disease due to Lupus Nephritis, was already taking the medication as part of her daily therapy. In the second patient, the drug was introduced at the very beginning of the contagion and then withdrawn. All of the home managed patients recovered completely without sequelae. Before returning to the outpatient clinic, all patients were checked with a nasal swab. All had cleared the virus.

#### 3.1.5. Multivariate Analysis

With the aim to identify the most impacting factors influencing the probability of death during the infection, the most significant variables were included in a multivariate regression analysis model (dependent variables: age, length of disease, cough, dyspnea, and AKI and CNI reduction; independent variable: death).

In the overall cohort, AKI development (*p* = 0.006 OR 50.4 CI 95% 3.0–836) and age (*p* = 0.04 OR 1.1 CI 95% 1.0–1.2) were the most important factors influencing the probability of death during the infection. (Table 4).

## 4. Discussion

Critical Coronavirus disease (COVID-19) has been described as a potentially life-threatening infection in some high-risk individuals such as the elderly and patients with several comorbid conditions or reduced immune-surveillance, such as patients with a bone marrow or solid organ transplants. Among kidney transplant recipients, the mortality rate varies between 24% and 30 % according to the series cited [7,8,9,10]. 

We report our single centre experience with 82 kidney transplanted patients with the COVID-19 infection, in two settings: hospitalized and home managed in two different outbreaks the last year. The incidence of infection in our transplant centre was 5.2%. This incidence—which is not as high as expected—could reflect the strict preventive measures adopted and our patients’ compliance, but may underestimate real infection prevalence among asymptomatic or paucisymptomatic patients. In fact, it is possible that minor symptoms (such as low-grade fever and malaise) were not even noticed or reported.

Cumulative mortality was 13.4%: 22.2% during the first outbreak and 9% during the second one. Patients who died presented a more serious infection together with several comorbid conditions: hypertension, heart disease, diabetes, chronic pulmonary obstructive disease, and cancer. As far as infection treatment is concerned, we acknowledge that during the first outbreak, pharmacological treatment was more empirical than evidence based; this explains the heterogeneous treatment adopted, also including the lack of consensus about immunosuppressive adjustment. In the most critically ill patients, we always advised to withdraw Mycophenolate, reduce or withdraw CNI, and increase the steroid dosage. However, in many cases, it was not up to us to decide. A reduction in mortality during the second outbreak is possibly related to better management of both the infection and immunosuppressive therapy. In particular, all hospitalized patients were treated with dexamethasone, which allowed us also to be more confident in reducing CNI therapy in the most compromised patients. We believe that both the improvement of patient management and the introduction of guidelines (between the first and second outbreak) had an impact on the outcome. This is demonstrated by the fact that although we had a two-fold increase of patients during the second outbreak, the total number of deaths remained the same, reducing the death rate.

Multivariate analysis demonstrated that the presence of AKI is a prognostic unfavourable variable for the outcome. COVID-19 has a direct renal invasion leading to glomerular and tubular damage and AKI [11]. AKI could be secondary to several factors, including a direct viral damage and an immune-mediated damage: the so-called cytokine storm [12]. The incidence of AKI in the general population has been reported in 5.1% up to 29 of cases according to age [13,14]. In our series, AKI cumulative incidence was 13.4% (11 patients): 22.2% in the first outbreak (6 patients) and 12% in the second (5 patients). However, evolution was dramatically different. During the first outbreak, only one patient recovered to basal creatinine level, while five died. In the second outbreak, one patient died and one started regular haemodialysis while three recovered completely. The incidence of AKI is higher in patients with established chronic kidney disease stage 1–3 and COVID-19 infection [12]. Transplanted patients have a further increased risk secondary to pharmacological immunosuppression. However, in this particularly severe infection, we believe that AKI could also be considered part of a multi-organ failure. AKI development has to be considered as another factor with a negative impact on the outcome. Acknowledging the small sample size, we cannot exclude that improved management immunosuppression may have also played an important role in reducing this severe complication [15,16].

Age was an independent risk factor for mortality. Hospitalized patients who died were about 10 years older than those who were non-hospitalized and had a longer transplant vintage that unexpectedly did not reach statistical significance. Older age and longer immunosuppressive treatment may have acted as a pre-disposing factor for the development of a more severe infection. However, unlike what is reported in the general population, age and associated comorbid conditions fail to reach statistical significance [17,18].

Finally, Zhou et al. demonstrated that some symptoms may be associated with the stage of the disease, but the duration of symptoms cannot be used to predict the outcome. In our analysis, duration of the disease did not correlate with the outcome but was significantly higher in hospitalized patients (*p* = 0.04), clearly indicating a longer disease in the most severely ill patients.

In our experience, 57% of patients had a mild infection that could be managed at home. We have not been able to identify any significant factor predicting either a better outcome or a less aggressive infection, but this requires a larger cohort of non-hospitalized COVID-19 positive patients. For this cohort, the following has been of paramount importance: avoidance of hospital attention, a persistent fever but with mild and not evolving respiratory symptoms, and the maintenance of a constant phone contact with the transplant physician.

## 5. Limitations

The first limitation that we acknowledge is the relatively small sample size. However, this is a single centre experience and we report on the cases that have come to our attention. This small number can also be related to the small number of nasopharyngeal swab performed, thus underestimating the real incidence of the COVID 19 infection. The second limitation is the non-homogeneous treatment received by hospitalized patients during the first outbreak, which has made understanding the impact on the outcome very difficult. Being that the patients hospitalized in different places, it was impossible for us to decide the treatment different from the modifications of the immunosuppressive therapy and to monitor COVID specific treatments. However, to the best of our knowledge, our cohort is one of the largest and is (so far) the only one reporting characteristics and outcomes in not critically ill kidney transplanted patients.

## 6. Conclusions

In conclusion, probably thanks to the preventive measures implemented at the very beginning of the COVID-19 pandemic, we report a lower percentage of infection than expected in a high-risk population such as kidney transplanted patients. Unfortunately, the incidence of death remains significantly higher than in the general population, although the odds have improved with the more recent therapy protocols.

## Figures and Tables

**Table 1:** Patients’ Characteristics Before the COVID-19 Infection.

Variable	
Number of patients (n)	82
Gender (n) (M/F)	54/28
Median age (years)	55 (46–46)
Type of transplant, n (deceased donor/living donor)	65/17
Median transplant vintage (months)	118 (39–229)
Steroids; CyA-Tac; MMF-MPA; mTor inhibitors (n)	74; 18–63; 55; 6
iRAS therapy (n/%)	21/26
Relevant comorbid conditions	N/%
Hypertension	67/82
Diabetes	9/32.1
Heart diseases/peripheral vascular disease	18/22
Active Neoplasia	2/2
Previous Neoplasia	12/14.3
Median laboratory values	
s-Creatinine (mg/dL)	1.46 (1.2–1.8)
Hemoglobin (g/dL)	12.9 (11.7–13.7)
serum-Albumin (g/dL)	4.2 (3.9–4.5)
Prot-U (g/24 h)	0.16 (0.10–0.43)

CyA: Cyclosporine; Tac: Tacrolimus; MMF-MPA: Mycophenolate; iRAS: Inhibitors of renin-angiotensin system; Prot-U: daily urinary pretein excretion; Continuous variables are reported as median [25%ils–75%ils].

**Table 2:** Principal Differences between Hospitalized and Non-Hospitalized Patients.

Variable	Hospitalized n = 37	Not Hospitalized = 45	*p*
Gender (n) (M/F)	26/11	28/17	0.48
Median age (years)	60 ± 11	50 ± 14	**0.001**
Median transplant vintage (months)	123 (6–341)	96 (7–346)	0.57
**Immunosuppressive therapy (n/%)**			
Steroids	34/91	40/88	0.72
CNI	36/97	44/97	0.10
MMF/MPA	24/64	36/80	0.61
mTor inhibitors	2/5	31/68	0.68

CNI: Calcineurin inhibitors MMF-MPA: Mycophenolate. Bold characters are used for significant results.

**Table 3:** Clinical and Outcome Differences between Hospitalized and Non-Hospitalized Patients.

	Hospitalized n = 37	Not Hospitalized = 45	*p*
**COVID-19 RELATED SYMPTOMS**			
Fever n/%	34/91.9	32/71.1	**0.04**
Cough n/%	24/64.8	11/ 24.4	**0.03**
Dyspnea n/%	23/62.1	4/8.8	**<0.0001**
Myalgia n/%	6/16.2	4/8.8	0.8
Diarrhea n/%	2/5.4	3/6.6	0.82
Dysgeusia and/or Anosmia n/%	1/2.7	9/20	**0.041**
AKI n/%	11/29.7	0/0	**0.001**
Length of disease (mean ± SD)	30 ± 28	21 ± 10	**0.04**
**OUTCOMES**			
Deaths n/%	11/29.7	0/0	**0.001**
Healings n/%	26/70.3	45/100	**0.001**

Bold characters are used for significant results.

**Table 4:** Multivariate Analysis Model.

Variable	OR	CI	*p*
**Age**	1.09	1.0026–1.2051	**0.04**
**AKI**	50.4	3.0462–836.0288	**0.006**
**Length of disease (days)**	0.95	0.9080–1.0073	0.09
**Cough**	1.54	0.1954–12.2283	0.67
**Dyspnea**	5.93	0.5097–69.0660	0.15
**CNI reduction**	0.06	0.0016–2.8822	0.16

AKI: acute kidney injury; CNI: calcineurine inhibitors. Bold characters are used for significant variables.

## Data Availability

If needed, data are available in anonymous form.
